# Association of High Calcitriol Serum Levels and Its Hydroxylation Efficiency Ratio with Disease Risk in SLE Patients with Vitamin D Deficiency

**DOI:** 10.1155/2021/2808613

**Published:** 2021-12-31

**Authors:** Mónica R. Meza-Meza, José Francisco Muñoz-Valle, Adolfo I. Ruiz-Ballesteros, Barbara Vizmanos-Lamotte, Isela Parra-Rojas, Erika Martínez-López, Edith Oregon-Romero, Yolanda Fabiola Márquez-Sandoval, Sergio Cerpa-Cruz, Ulises de la Cruz-Mosso

**Affiliations:** ^1^Proyecto Inmunonutrición y Genómica Nutricional en las Enfermedades Autoinmunes, Centro Universitario de Ciencias de la Salud, Universidad de Guadalajara, Guadalajara, Jalisco 44340, Mexico; ^2^Instituto de Nutrigenética y Nutrigenómica Traslacional, Centro Universitario de Ciencias de la Salud, Universidad de Guadalajara, Guadalajara, Jalisco 44340, Mexico; ^3^Instituto de Investigación en Ciencias Biomédicas, Centro Universitario de Ciencias de la Salud, Universidad de Guadalajara, Guadalajara, Jalisco 44340, Mexico; ^4^Laboratorio de Investigación en Obesidad y Diabetes, Facultad de Ciencias Químico-Biológicas, Universidad Autónoma de Guerrero, Chilpancingo de los Bravo, Guerrero 39087, Mexico; ^5^Departamento de Reumatología, O.P.D. Hospital Civil de Guadalajara Fray Antonio Alcalde, Guadalajara, Jalisco 44280, Mexico

## Abstract

Vitamin D (calcidiol) deficiency in systemic lupus erythematosus (SLE) is more frequent than in healthy subjects (HS); it is associated with clinical activity and damage in SLE. Although calcidiol is considered the best indicator of the vitamin D serum status, its deficiency could not reflect its hydroxylation efficiency ratio and calcitriol serum status. This study was aimed at assessing the association of calcidiol and calcitriol serum levels and its hydroxylation efficiency ratio with the risk to clinical and renal disease activities in SLE patients. A cross-sectional study was conducted in 308 SLE and HS women; calcidiol and calcitriol serum levels were evaluated by immunoassays. SLE patients showed lower calcidiol serum levels *vs.* HS (21.2 *vs.* 24.2 ng/mL; *p* < 0.001). Active SLE patients presented higher calcidiol/calcitriol ratio scores *vs.* inactive SLE patients (2.78 *vs.* 1.92 pg/ng; *p* = 0.02), and SLE patients with renal disease activity showed a pattern of calcidiol-deficient levels (19.5 *vs.* 25.3 ng/mL; *p* < 0.04) with higher calcitriol levels (47 pg/mL *vs.* 41.5 pg/mL; *p* = 0.02) and calcidiol/calcitriol ratio scores (2.13 *vs.* 1.54 pg/ng; *p* < 0.02) compared to SLE patients without renal disease activity. Calcidiol levels were negatively correlated with calcitriol levels (*r* = −0.26; *p* = 0.001) and urine proteins (mg/dL) (*r* = −0.39; *p* < 0.01). Regarding calcitriol levels, it was positively correlated with the blood lymphocyte count (*r* = 0.30; *p* < 0.001) and negatively correlated with the glomerular filtration rate (*r* = −0.28; *p* = 0.001). Moreover, the calcitriol/calcidiol ratio was positively correlated with urine proteins (*r* = 0.38; *p* < 0.01). The calcidiol deficiency (OR = 2.27; 95% CI = 1.15-4.49; *p* < 0.01), high calcitriol levels (*T*3^rd^, OR = 4.19, 95% CI = 2.23-7.90; *p* < 0.001), and a high calcitriol/calcidiol ratio score (*T*3^rd^, OR = 5.93, 95% CI: 3.08-11.5; *p* < 0.001) were associated with the risk for SLE. In conclusion, a pattern of calcidiol deficiency with high calcitriol serum levels and a high vitamin D hydroxylation efficiency ratio was associated with disease risk in SLE patients.

## 1. Introduction

Systemic lupus erythematosus (SLE) is a chronic autoimmune disease characterized by tolerance loss to nuclear self-antigens, autoantibody production, damage organs, and vitamin D deficiency [1–3]. The substantial reduction in clearance activity and formation of immune complexes may lead to local inflammation and damage with an aberrant expression of autoreactive T helper (Th) cells and proinflammatory cytokines, which are critical factors associated with SLE pathogenicity and lupus nephritis (LN) development [4, 5].

Several studies suggest that SLE is a Th type 2 (Th2) profile-driven disease. However, in SLE, different lymphocyte subsets drives its pathophysiology. The proinflammatory Th1, Th2, and Th17 profiles positively correlated with high clinical disease activity and were described as elevated in humans and murine models [4, 6]. The contributions of Th2 response to renal disease activity and LN development include the IL-6 and IL-4 production by activated basophils, which leads to autoantibody deposition in the kidney, increasing in a positive feedback loop the Th2 response, and B cell activation [4].

Currently, it has been described that the nutrients could play a crucial role in the survival, proliferation, and activation of immune cells. Vitamin D is a widely studied candidate nutrient in pathologies with an immune component [7, 8]. It can be obtained from the endogenous synthesis in the epidermis by exposure to UVB light and from foods and supplements in the form of ergocalciferol (vitamin D_2_) and cholecalciferol (vitamin D_3_) [9].

The main metabolite used to measure the vitamin D serum status is calcidiol [25 (OH)D]. However, its active metabolite calcitriol [1*α*,25(OH)_2_D] exerts its effects through genomic mechanisms modulated by the nuclear vitamin D receptor (VDR)/retinoid X receptor (RXR) complex to bind to vitamin D response elements (VDRE) in target genes of several immune cells [9]. Hence, calcitriol is considered an immunomodulator that participates in the control of self-tolerance and influences immune cell differentiation and production of several cytokines [1, 8, 10].

Calcitriol suppresses the differentiation of Th1 cells while promoting the formation of tolerogenic dendritic cells, T regulatory cells (Tregs), Th2 differentiation, IL-4 production, the anti-inflammatory status, and reduction of autoantibodies [7, 11, 12]. Nevertheless, the effects of calcitriol on B cells that are pivotal in SLE are still unclear because it depends on its available quantity, cytokine microenvironment, activation, and differentiation status of B cells [11].

In a biphasic dose-dependent manner, high calcitriol concentrations stimulate the plasma cell development; when B cells are terminally differentiating, induce a high IL-10 production, B cell hyperactivity, and autoantibody production associated with high clinical disease activity in SLE [5, 11, 13], highlighting that vitamin D could be an immunomodulatory nutrient that plays a bimodal role during physiological and pathological events.

Notably, in SLE, vitamin D deficiency has been associated with high severity, progression, and comorbidity development such as LN [14–16]. Its deficiency in SLE patients is more frequent compared to healthy individuals [14, 17], with a prevalence rate of calcidiol insufficiency (<30 ng/mL) of 38–96% and deficiency (<20 ng/mL) of 3–35% in several populations [1, 7, 14–16, 18, 19].

Most studies in SLE patients have focused on evaluating the calcidiol serum levels with controversial findings of its relationship with the clinical and renal disease activities or comorbidities, and the association of calcidiol with the active metabolite calcitriol has not been previously reported in other studies conducted in SLE patients so far.

Therefore, the physiological complexity of the synthetic pathway of vitamin D suggests that the efficient regulation of vitamin D hydroxylation might be more crucial than the concentration of any D metabolite alone, which could be altered in SLE. Although calcidiol is considered the best indicator of the serum vitamin D status, its deficiency could not reflect calcitriol's serum status. According to this, the calcitriol/calcidiol ratio assessment, which theoretically represents how many picograms of calcitriol are produced per nanogram of circulating calcidiol, could be considered representative of the vitamin D hydroxylation efficiency [20]. Based on these previous findings, the aim of our study was at assessing the association of calcidiol, calcitriol, and its hydroxylation efficiency ratio with the risk to clinical and renal disease activities in SLE patients.

## 2. Material and Methods

### 2.1. Participants

A cross-sectional study was conducted in 308 women from an unrelated Mexican-mestizo population, made up of two groups: the first group was made up of 157 SLE women patients classified according to the 1997 SLE American College of Rheumatology (ACR) [21], recruited between 2017 and 2020 from the Rheumatology Department of the *Hospital Civil Fray Antonio Alcalde*, Guadalajara, Jalisco, Mexico. SLE patients had no recent infections, trauma, surgery, pregnancy, or systemic autoimmune conditions unrelated to SLE.

The second group was composed of 151 healthy female subjects (HS) as a reference group, recruited from the same geographical area. The HS included did not refer to recent infections, trauma, surgery, pregnancy, or autoimmune conditions; they were also asked about the presence of autoimmune diseases in their families. They did not mention that their close relatives like siblings, parents, and grandparents presented autoimmune diseases. Both study groups presented an ancestry of at least back three generations in the same geographical region.

### 2.2. Ethical Considerations

Before enrollment in the study, all participants provided signed written informed consent. The Research Ethical Committee of the *Centro Universitario de Ciencias de la Salud*, *Universidad de Guadalajara* (CI-03419 CUCS-UdG), and the *Hospital Civil Fray Antonio Alcalde* (no. 280/19) approved the research study, according to the international ethical guidelines.

### 2.3. SLE Clinical Disease Activity and Damage Indexes

The clinical disease activity presented at enrollment in the study was evaluated by the Mexican Systemic Lupus Erythematosus Disease Activity Index (Mex-SLEDAI), which is a validated clinical disease activity index adjusted for the Mexican-mestizo population [22], and the disease damage was evaluated according to the Systemic Lupus International Collaborating Clinics-American College of Rheumatology Damage Index (SLICC-ACR DI) criteria [23].

### 2.4. Quantification of Vitamin D Metabolites

A blood sample was obtained from each patient from antecubital venipuncture, collected in the morning after an overnight fast (12 h), and then, centrifuged for 10 min to obtain the serum. Calcidiol and calcitriol serum levels were determined using an ELISA plate reader (Multiskan GO, Thermo Scientific™ 51119000, USA) with commercial competitive ELISA assays, according to the manufacturer's instructions. For the quantification of calcidiol (25-hydroxy-vitamin D), the human soluble 25-OH Vitamin D ELISA Kit (detection limit of 1.6 ng/mL, Eagle Biosciences®, VID31-K01, USA) was used and the calcitriol serum quantification (1,25*α*-dihydroxyvitamin D_3_) was by the human soluble 1,25*α*(OH)_2_D_3_ ELISA kit (sensitivity < 0.10 pg/mL, Cusabio®, CSB-E0512h, China).

### 2.5. Classification Criteria and Definitions

The reference cutoff values for the interpretation of serum calcidiol levels were (a) deficiency (<20 ng/mL), (b) insufficiency (≥20 to <30 ng/mL), and (c) sufficiency (≥30 ng/mL), according to cutoff values reported [24, 25].

For calcitriol, the reference cutoff values were 15–60 pg/mL [26, 27]. However, for the evaluated sample, we stratified the calcitriol levels classified into tertiles: (a) low calcitriol serum levels = *T*1^st^ (0.33 to <33.6 pg/mL), (b) average calcitriol serum levels = *T*2^nd^ (≥33.6 to <48.7 pg/mL), and (c) high calcitriol serum levels = *T*3^rd^ (≥48.7 to 157.3 pg/mL).

To estimate the vitamin D hydroxylation efficiency, we calculated the calcitriol/calcidiol ratio based on values of calcitriol (pg/mL) and calcidiol (ng/mL), which resulted in arbitrary units (pg/ng) that should indicate how many pg of calcitriol is produced per ng of circulating calcidiol [20]. The calcitriol/calcidiol ratio score was also stratified into tertiles: (a) low conversion rate of calcidiol to calcitriol = *T*1^st^ (0.01 to <1.36 pg/ng), (b) average conversion rate of calcidiol to calcitriol = *T*2^nd^ (≥1.36 to ≤2.23 pg/ng), and (c) high conversion rate of calcidiol to calcitriol = *T*3^rd^ (≥2.23 to 23.6 pg/ng).

Also, the glomerular filtration rate (GFR) was estimated with the Chronic Kidney Disease Epidemiology Collaboration (CKD-EPI) equation based on serum creatinine (mg/dL) and using the parameters sex, race, and age, expressed in mL/min/1.73 m^2^ of the body surface area [28]. The GFR (mL/min/1.73m^2^) was classified by the Kidney Disease: Improving Global Outcomes 2012 Clinical Practice Guideline (KDIGO 2012) categories: (a) G1 (normal or high ≥ 90), (b) G2 (mildly decreased: 60–89), (c) G3a (mildly to moderately decreased: 45-59), (d) G3b (moderately to severely decreased: 30–44), (e) G4 (severely decreased: 15–29), and (f) G5 (kidney failure < 15) [29].

### 2.6. Statistical Analysis

The statistical analyses were performed with the software STATA v 15 (College Station, TX, USA) and GraphPad Prism v 8.0 (San Diego, CA, USA). The statistical power was evaluated according to the calculation of the sample size, performed with an estimated error margin of 2% with a confidence degree of 95% and an expected prevalence of serum vitamin D insufficiency and deficiency excess in SLE patients of 83–96% and 3.1–4.6%, respectively, reported in a previous study in Mexican SLE patients [30].

The Shapiro–Wilk test was used to determine the nonparametric and parametric distribution of the continuous variables. For the descriptive analysis, the nominal discontinuous variables were expressed as frequencies; the continuous variables with parametric distribution were expressed as means ± standard deviation (SD) and the nonparametric variables as medians and percentiles 5^th^–95^th^. For the inferential analysis, the *χ*^2^ test was used to compare proportions. For parametric quantitative determinations of two groups, Student's *t*-test was used and the Mann–Whitney *U* test was used for nonparametric quantitative determinations. We used linear and logistic regression models to evaluate the association and contribution of the calcidiol and calcitriol serum levels as well as the calcitriol/calcidiol ratio to the clinical and renal disease activities. The differences were considered significant with a *p* value < 0.05.

## 3. Results

### 3.1. Vitamin D Serum Metabolite Levels in SLE Patients and Healthy Subjects

A total of 157 female SLE patients were evaluated with a median age of 37 (20–59) years old. According to the Mex-SLEDAI score, 57% of SLE patients were without clinical disease activity (Mex-SLEDAI < 2) and 45% were with clinical disease activity (Mex-SLEDAI ≥ 2). As a reference healthy group representative of the same population, a total of 151 HS women were evaluated with a median age of 30 (19–59) years old.

**Figure 1:**
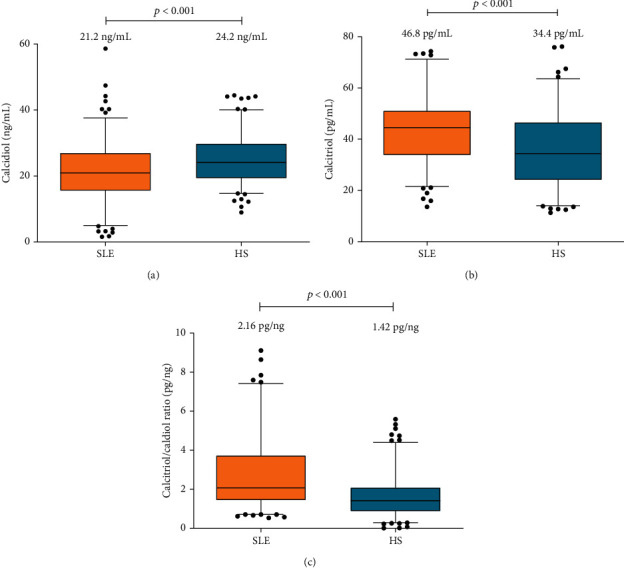
Vitamin D serum metabolites and vitamin D hydroxylation efficiency ratio from SLE patients *vs.* HS. (a) Calcidiol serum levels of SLE patients (5.1–40.4 ng/mL) *vs.* HS (14.8–40.2 ng/mL); (b) calcitriol serum levels of SLE patients (22.1–103 pg/mL) *vs.* HS (8.92–89.8 pg/mL); (c) efficiency of vitamin D hydroxylation by the calcitriol/calcidiol ratio of SLE patients (0.73–10.5 pg/ng) *vs.* HS (0.30–4.31 pg/ng). Data provided in median (*p*5^th^–*p*95^th^ in parenthesis); Mann–Whitney *U* test. SLE: systemic lupus erythematosus; HS: healthy subjects.

SLE patients showed lower calcidiol serum levels compared to HS (SLE = 21.2*vs.*HS = 24.2 ng/mL; *p* < 0.001), both classified as calcidiol insufficiency (20 to <30 ng/mL) (Figure 1(a)). According to the calcidiol reference values, a higher frequency of SLE patients showed calcidiol deficiency (<20 ng/mL) (SLE = 45%*vs.*HS = 27%), followed by calcidiol insufficiency (≥20 to <30 ng/mL) (SLE = 38%*vs.*HS = 49%), and low calcidiol sufficiency frequency (≥30 ng/mL) (SLE = 17%*vs.*HS = 24%) (*p* < 0.01) (data not shown).

Regarding calcitriol serum levels, SLE patients presented higher values with 46.8 pg/mL *vs.* 34.4 pg/mL in HS (*p* < 0.001) (Figure 1(b)). The calcitriol serum levels were categorized in tertiles, and we observed in the SLE patients a higher frequency in the third tertile classified as high calcitriol serum levels (*T*3^rd^ = 43%), followed by the second tertile with the average calcitriol levels (*T*2^nd^ = 37%), and the first tertile with the low calcitriol levels (*T*1^st^ = 20%), compared with HS who presented a higher frequency in the first tertile (*T*1^st^ = 47%), followed in decreasing order by the *T*2^nd^ (30%), and the *T*3^rd^ (24%) (*p* < 0.001) (data not shown).

The vitamin D hydroxylation efficiency was estimated by the calcitriol/calcidiol ratio, and the SLE patients showed a higher score of 2.16 pg/ng, interpreted as a high-calcidiol-to-calcitriol conversion rate compared to HS (1.42 pg/ng) (*p* < 0.001) (Figure 1(c)).

### 3.2. Vitamin D Serum Levels Stratified by Clinical Disease Activity

To evaluate the association of calcidiol serum levels with the clinical disease activity, the SLE patients were stratified according to the Mex-SLEDAI score in inactive SLE patients (Mex-SLEDAI < 2) and active SLE patients (Mex-SLEDAI ≥ 2). Active SLE patients showed a higher frequency of renal disease activity (45%; *p* = 0.01) than inactive SLE patients (Table 1). A trend of lower calcidiol serum levels (21.2 ng/mL; *p* = 0.14) and a higher frequency of calcidiol deficiency (48%; *p* = 0.34) was observed in active SLE patients in comparison to inactive SLE patients (Table 1). No differences were observed on calcitriol serum levels between active and inactive SLE patients. However, active SLE patients showed a trend of higher frequency of calcitriol serum levels in the third tertile (*T*3^rd^ = 50%) compared to inactive SLE patients (*p* = 0.26) (Table 1).

About the calcitriol/calcidiol ratio, the active SLE patients showed a higher calcitriol/calcidiol ratio (2.78 pg/ng; *p* = 0.02) and lower serum calcium levels (8.94 mg/dL; (*p* = 0.01) compared to inactive SLE patients (Table 1).

### 3.3. Vitamin D Serum Levels in SLE Patients with Renal Disease Activity

Clinical characteristics and vitamin D metabolite serum levels were evaluated in a subgroup of SLE patients according to the presence or absence of renal disease activity. About their clinical characteristics, SLE patients with renal disease activity showed higher values of the damage score (SLICC = 1; *p* = 0.02) and clinical disease activity score (Mex-SLEDAI = 2; *p* < 0.01) compared to SLE without renal disease activity (Table 2). Additionally, the SLE patients with renal disease activity showed higher frequency of proteinuria (48% *vs.* 3%; *p* < 0.001), microalbuminuria (79% *vs.* 21%; *p* = 0.001), hematuria (80% *vs.* 22%; *p* < 0.001), and pyuria (90% *vs.* 61%; *p* = 0.02) in comparison with SLE patients without renal disease activity (data not shown).

**Figure 2:**
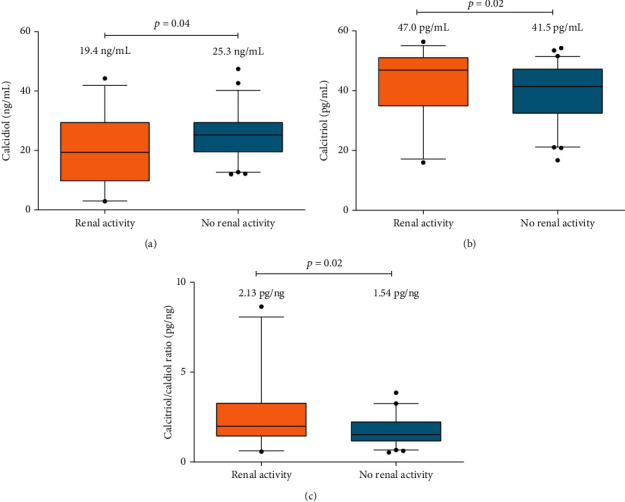
Vitamin D serum metabolites and vitamin D hydroxylation efficiency ratio from SLE patients stratified by renal disease activity. (a) Calcidiol serum levels (ng/mL) from SLE patients with renal activity (3.26–36.6 ng/mL) and no renal activity (12.7–40.4 ng/mL); (b) calcitriol serum levels (pg/mL) from SLE patients with renal activity (19.0–53.2 pg/mL) and no renal activity (21.8–51.2 pg/mL); (c) calcitriol/calcidiol ratio (pg/ng) from SLE patients with renal activity (0.76–15.3 pg/ng) and no renal activity (0.71–3.21 pg/ng). Data provided in median (*p*5^th^–*p*95^th^ in parenthesis), Mann–Whitney test. SLE: systemic lupus erythematosus.

Regarding the evaluation of vitamin D metabolites, SLE patients with renal disease activity showed lower calcidiol serum levels (19.5 ng/mL) classified as calcidiol deficiency in comparison to SLE patients without renal disease activity (25.3 ng/mL) classified as insufficiency (*p* < 0.04) (Figure 2(a)). Moreover, higher calcidiol deficiency was observed in SLE patients with renal disease activity (52%) compared to SLE patients without renal disease activity (25%) (*p* = 0.03) (Table 2). By contrast, SLE patients with renal disease activity showed higher calcitriol levels (47 pg/mL) (*p* = 0.02) (Figure 2(b)) and a higher frequency of high calcitriol levels in the third tertile (*T*3^rd^: 41%) (*p* = 0.03) compared to SLE patients without renal activity (Table 2).

About the calcitriol/calcidiol ratio, SLE patients with renal disease activity showed a higher conversion rate of calcidiol to calcitriol (2.13 pg/ng), compared to SLE patients without renal disease activity (1.54 pg/ng; *p* < 0.02) (Figure 2(c)).

### 3.4. Calcitriol Serum Levels and Vitamin D Hydroxylation Efficiency Ratio Stratified by the Calcidiol Reference Values

Based on the previous result, we evaluated the calcitriol serum levels and the calcitriol/calcidiol ratio pattern according to the calcidiol reference values stratified in deficiency, insufficiency, and sufficiency of vitamin D.

**Figure 3:**
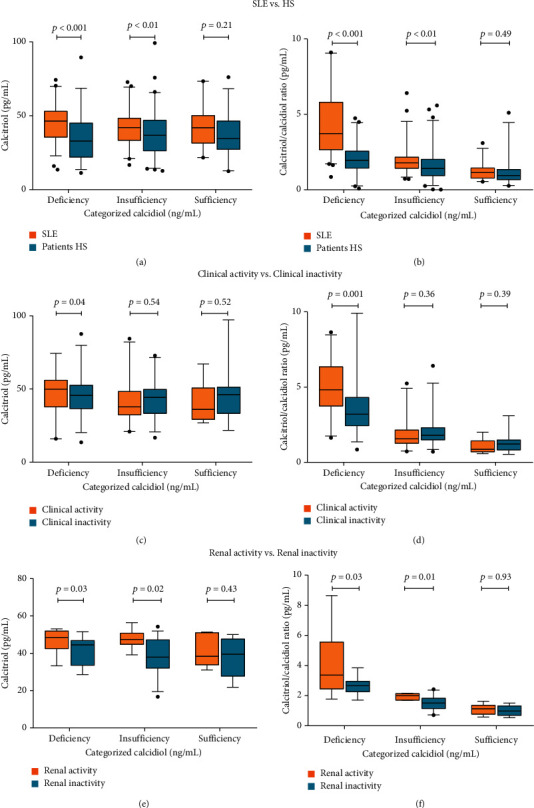
Calcitriol serum levels and vitamin D hydroxylation efficiency ratio stratified by the calcidiol reference values. (a) Calcitriol serum levels (pg/mL) from SLE patients *vs.* HS, (b) Efficiency of vitamin D hydroxylation (pg/ng) from SLE patients *vs.* HS; (c) calcitriol serum levels (pg/mL) from SLE patients with clinical activity *vs.* SLE patients with clinical inactivity; (d) efficiency of vitamin D hydroxylation (pg/ng) from SLE patients with clinical activity *vs.* SLE patients with clinical inactivity; (e) calcitriol serum levels (pg/mL) from SLE patients with renal activity *vs.* SLE patients with no renal activity, (f) efficiency of vitamin D hydroxylation (pg/ng) from SLE patients with renal activity *vs.* SLE patients with no renal activity. Data provided in median, Mann–Whitney test. Calcidiol reference values: deficiency (<20 ng/mL), insufficiency (≥20 to <30 ng/mL), and sufficiency (≥30 ng/mL).

We observed significant differences with higher calcitriol serum levels in SLE patients *vs.* HS with calcidiol deficiency (SLE = 51.7 pg/mL [32.6–109] *vs.*HS = 32.7 pg/mL [7.84–64.8; *p* < 0.001), followed by the calcidiol insufficiency group (SLE = 43.9 pg/mL [21.1–97.5] *vs.*HS = 36.7 pg/mL [7.94–102]; *p* < 0.01), with similar calcitriol values in a calcidiol sufficiency condition (SLE = 44.5 pg/mL*vs.*HS = 34.9 pg/mL; *p* = 0.20). Notably, a pattern of decreasing calcitriol serum levels as calcidiol sufficiency was achieved in both study groups was observed (Figure 3(a)).

About the efficiency of vitamin D hydroxylation, SLE patients showed a higher calcidiol to calcitriol conversion compared to HS in the range of calcidiol deficiency (SLE = 4.02 pg/ng [1.78–18] *vs.*HS = 1.95 pg/ng [0.43–4.01]; *p* < 0.001), followed in descending order by the calcidiol insufficiency status (SLE = 1.79 pg/ng [0.84–4.51] *vs.*HS = 1.42 pg/ng [0.30–4.53]; *p* < 0.01), while in the calcidiol sufficiency range, SLE patients and HS showed a similar calcitriol/calcidiol ratio (SLE = 1.14 pg/ng*vs.*HS = 0.95 pg/ng; *p* = 0.49). This pattern highlights that in a serum calcidiol deficiency, the conversion rate of calcidiol to calcitriol increases and the conversion decreases as sufficiency is achieved (Figure 3(b)).

Additionally, when we compare the SLE patients according to the presence of clinical disease activity and stratified by the calcidiol reference values, we observed higher calcitriol serum levels in active SLE patients with calcidiol deficiency (clinical disease activity = 54.9 pg/mL [23.7–131] *vs.* clinical disease inactivity = 46.5 pg/mL [23.4–98.4]; *p* = 0.04) and no differences between calcitriol were observed in the comparison to active *vs.* inactive SLE patients in the ranges of calcidiol insufficiency (clinical disease activity = 38.1 pg/mL*vs.* clinical disease inactivity = 45.4 pg/mL; *p* = 0.54), and calcidiol sufficiency (clinical disease activity = 36.2 pg/mL*vs.* clinical disease inactivity = 46.2 pg/mL; *p* = 0.52) (Figure 3(c)).

With respect to the vitamin D hydroxylation efficiency, we observed a higher calcitriol/calcidiol ratio in active SLE patients (clinical disease activity = 5.56 pg/ng [1.90–22.5] *vs.* clinical disease inactivity = 3.20 pg/ng [1.40–1.43]; *p* = 0.001), with a descending pattern of conversion of calcidiol to calcitriol with no significant differences in SLE patients with calcidiol insufficiency (clinical disease activity = 1.56 pg/ng*vs.* clinical disease inactivity = 1.80 pg/ng; *p* = 0.36) and with calcidiol sufficiency (clinical disease activity = 0.87 pg/ng*vs.* clinical disease inactivity = 1.22 pg/ng; *p* = 0.39). These results highlight that active SLE patients with calcidiol deficiency have higher calcitriol serum levels, with a high conversion rate of calcidiol to calcitriol compared to inactive SLE patients with a similar calcidiol deficiency (Figure 3(d)).

Because renal disease activity is one of the main variables contributing to the clinical disease activity, we made the same comparative analysis about the calcitriol serum levels and the vitamin D hydroxylation efficiency scores stratified by calcidiol serum reference values. We observed that SLE patients with renal disease activity and calcidiol deficiency showed higher calcitriol serum levels (renal disease activity = 48.7 pg/mL [33.3–53.2] *vs.* renal disease inactivity = 44.7 pg/mL [28.7–51.7]; *p* = 0.03) and the same pattern was observed in the range of calcidiol insufficiency (renal disease activity = 47.5 pg/mL [39.3–56.5] *vs.* renal disease inactivity = 38.1 pg/mL [19.5–52.1]; *p* = 0.02) in comparison with renal-inactive patients. This differential pattern was not observed in the comparison of renal active SLE versus renal inactive patients, both with calcidiol sufficiency (renal activity = 38.5 pg/mL*vs.* renal inactivity = 39.6 pg/mL; *p* = 0.42) (Figure 3(e)).

Concerning vitamin D hydroxylation efficiency, the SLE patients with renal disease activity and calcidiol deficiency showed a higher calcidiol to calcitriol conversion rate (renal disease activity = 3.73 pg/ng [1.78–17.1] *vs.* renal disease inactivity = 2.73 pg/ng [1.71–11.1]; *p* = 0.03), followed by a significantly similar pattern in a calcidiol insufficiency status (renal disease activity = 2.0 pg/ng [1.69–2.17] *vs.* renal disease inactivity = 1.51 pg/ng [0.72–2.38]; *p* = 0.01), whereas in renal active SLE patients and renal inactive patients, both with calcidiol sufficiency, no significant differences were observed (renal disease activity = 1.14 pg/ng*vs.* renal disease inactivity = 0.98; *p* = 0.93) (Figure 3(f)). This similar pattern observed in the comparison of SLE patients with renal activity shows that the calcidiol deficiency and insufficiency are related to higher calcitriol serum levels and higher conversion rate of calcidiol to calcitriol compared to renal inactive SLE patients.

### 3.5. Association of Vitamin D Metabolites and Efficiency of Vitamin D Hydroxylation with SLE Clinical Variables

**Figure 4:**
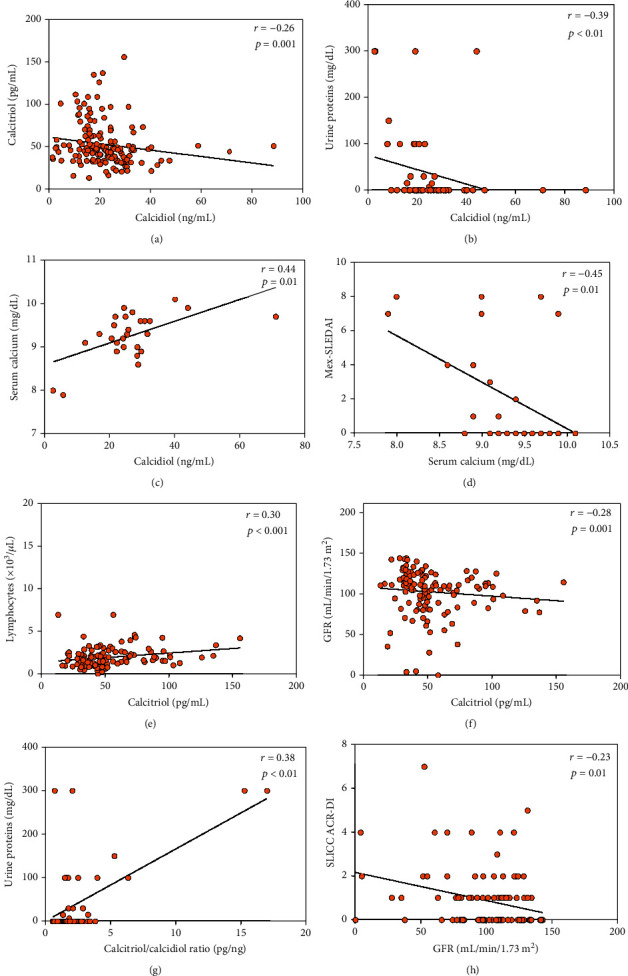
Correlations of vitamin D metabolite serum levels and vitamin D hydroxylation efficiency ratio with clinical and renal variables in SLE patients. (a) Correlation of calcidiol serum levels and calcitriol serum levels; (b) correlation of calcidiol serum levels and urine proteins; (c) correlation of calcidiol serum levels and serum calcium; (d) correlation of serum calcium and Mex-SLEDAI; (e) correlation of calcitriol serum levels with blood lymphocyte count; (f) correlation of calcitriol serum levels and GFR; (g) correlation of calcitriol/calcidiol ratio and urine proteins; (h) correlation of GFR with the SLICC ACR-DI score. *r*: Spearman's correlation coefficient; GFR: glomerular filtration rate.

About the vitamin D metabolites, we observed that calcidiol serum levels were negatively correlated with the calcitriol serum levels (*r* = −0.26; *p* = 0.001; Figure 4(a)), a finding that was not observed in HS. As well, the calcidiol serum levels were negatively correlated with urine proteins (mg/dL) (*r* = −0.39; *p* < 0.01; Figure 4(b)) and positively correlated with serum calcium (mg/dL) (*r* = 0.44; *p* = 0.01; Figure 4(c)). Notably, the serum calcium correlated negatively with the de Mex-SLEDAI index (*r* = −0.45; *p* = 0.01; Figure 4(d)).

Calcitriol serum levels were positively correlated with the blood lymphocyte count (*r* = 0.30; *p* < 0.001; Figure 4(e)) and negatively correlated with the GFR (*r* = −0.28; *p* = 0.001; Figure 4(f)). Moreover, the calcitriol/calcidiol ratio was positively correlated with urine proteins (*r* = 0.38; *p* < 0.01; Figure 4(g)) and the GFR was negatively correlated with the SLICC ACR-DI (*r* = −0.23; *p* = 0.01; Figure 4(h)).

Based on the findings observed, we estimated the association of vitamin D metabolites and the calcitriol/calcidiol ratio with the risk to SLE and clinical and renal disease activities, using multiple logistic and linear regression models.

**Figure 5:**
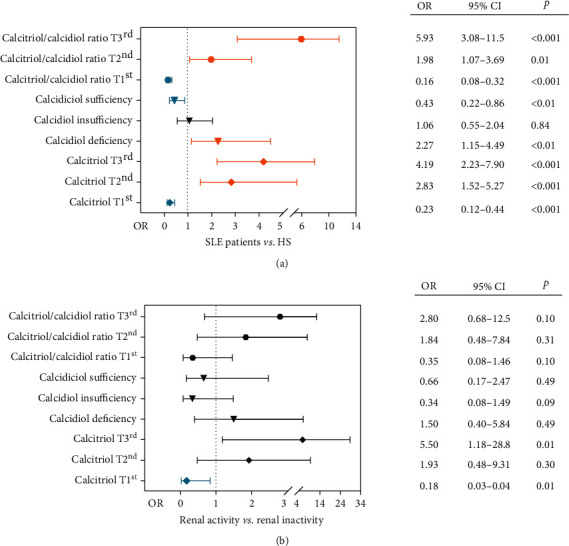
Association of vitamin D metabolite serum levels and vitamin D hydroxylation efficiency ratio with the SLE and renal disease activity. (a) Association of vitamin D metabolite serum levels and calcitriol/calcidiol ratio with the SLE (SLE patients *vs.* HS); (b) association of vitamin D metabolite serum levels and calcitriol/calcidiol ratio with the presence of renal activity. HS: healthy subjects. OR: odds ratio, Fisher's exact test, blue (protective OR < 1) and orange (risk OR ≥ 1) graphics, *p* values < 0.05, 95% confidence interval (CI). Black graphics: not significant differences (*p* > 0.05) or high confidence intervals. Calcitriol/calcidiol ratio tertiles: *T*3^rd^ (≥2.23 to 23.6 pg/ng), *T*2^nd^ (≥1.36 to ≤2.23 pg/ng), and *T*1^st^ (0.01 to <1.36 pg/ng). Calcidiol reference values: deficiency (<20 ng/mL), insufficiency (≥20 to <30 ng/mL), and sufficiency (≥30 ng/mL). Calcitriol tertiles: *T*3^rd^ (≥48.7 to 157.3 pg/mL), *T*2^nd^ (≥33.6 to <48.7 pg/mL), and *T*1^st^ (0.33 to <33.6 pg/mL).

Regarding calcidiol serum levels, we observed that SLE patients with calcidiol deficiency had a 2.27-fold higher risk for SLE (OR = 2.27; *p* < 0.01), while SLE patients with calcidiol serum levels within sufficiency values have a 2.32-fold lower risk for SLE (OR = 0.43; *p* < 0.01) (Figure 5(a)).

About calcitriol serum levels, the SLE patients with calcitriol serum levels within the first tertile had a 4.34-fold lower risk for SLE (*T*1^st^, OR = 0.23; *p* < 0.001), while the SLE patients with calcitriol levels within the second tertile had 2.83-fold higher risk for the disease (*T*2^nd^, OR = 2.83; *p* < 0.001) and those within the third tertile had 4.19-fold higher risk to SLE (*T*3^rd^, OR = 4.19; *p* < 0.001) (Figure 5(a)).

Concerning the efficiency of vitamin D hydroxylation assessed by the calcitriol/calcidiol ratio, SLE patients with a low-efficiency rate of vitamin D hydroxylation (first tertile) had 6.25-fold lower risk for SLE (*T*1^st^; OR = 0.16; *p* < 0.001), while the SLE patients with an average efficiency rate of vitamin D hydroxylation (second tertile), who had 1.98-fold higher risk for SLE (*T*2^nd^, OR = 1.98; *p* = 0.01), and those with a high calcitriol/calcidiol ratio (third tertile) had 5.93-fold higher risk for SLE (*T*3^rd^, OR = 5.93; *p* < 0.001) (Figure 5(a)).

Besides, the SLE presence also was associated to low calcidiol serum levels (*β* coefficient = −3.6; *R*^2^ = 0.02; *p* = 0.01), with high calcitriol serum levels (*β* coefficient = 14.4; *R*^2^ = 0.08; *p* < 0.001), and a high calcidiol to calcitriol conversion rate (*β* coefficient = 1.93; *R*^2^ = 0.07; *p* < 0.001) (data not shown).

Notably, SLE patients with low calcitriol serum values within the first tertile had 5.55-fold lower risk to renal disease activity (OR = 0.18; *p* = 0.01) (Figure 5(b)). Finally, we observed that the presence of clinical and renal disease activities in SLE patients was associated with a significant high calcidiol to calcitriol conversion rate (*β* coefficient = 1.67, *R*^2^ = 0.04, *p* = 0.01 and *β* coefficient = 1.76, *R*^2^ = 0.09, *p* < 0.01, respectively) (data not shown).

## 4. Discussion

SLE patients have been reported to have a higher susceptibility to present vitamin D deficiency than the general population. We observed a higher frequency of calcidiol deficiency in SLE patients *vs.* HS, similar to other cross-sectional studies that reported lower calcidiol serum levels in SLE patients compared to HS [15, 31]. However, the prevalence of calcidiol deficiency varies from 3% to 67% in studies conducted in different SLE populations; these wide ranges in the differences in the calcidiol deficiency frequency observed between populations could be influenced by environmental and genetic factors that could modify the calcidiol serum levels: such as geographic latitude; low UV light exposure time; pharmacotherapy used such as glucocorticoids, which have a negative effect on vitamin D serum levels; additional comorbidities presented such as obesity, hypothyroidism, and renal damage; genetic polymorphisms such as those described in the *VDR* gene; older age of the participants included; and heterogeneous clinical disease activity status that presented the SLE patients in each study [7, 15, 18, 31].

Calcidiol deficiency in SLE patients has been associated with clinical disease activity and damage, with controversial findings [31–33]. In our study, no association between calcidiol levels with the clinical disease activity was observed; however, there was a trend of lower calcidiol serum values in active SLE patients and calcidiol serum levels were negatively correlated with calcitriol; this correlation pattern was not observed in HS. Notably, calcitriol serum levels were higher in the SLE patients compared to HS and high calcitriol serum levels in SLE patients were associated with the risk to the disease. Recently, very few studies in the literature have evaluated the calcitriol serum levels in SLE, despite being the biologically active metabolite of vitamin D [34]. However, it has been described that in a serum calcidiol-deficient status, calcitriol levels are often elevated due to local calcitriol synthesis in several organs and tissues to regulate the genes related to cellular proliferation and other homeostatic healthy important functions [24]. High calcitriol levels in pathological conditions could be indicative of active inflammatory disease; in a study of patients with granulomatous diseases, high calcitriol production by inflammatory cells has been associated with the inflammatory process, and in another study in patients with early diagnoses of tuberculous pleuritis, elevated calcitriol levels have been associated with active pulmonary tuberculosis [35].

Although similar data have not been reported in SLE or patients with other autoimmune diseases, high calcitriol serum levels have been described as a risk factor in other diseases such as cancer and allergies [35–38]. The antitumor properties of calcitriol have been reported in several studies; however, the calcitriol excess may not always be beneficial, particularly in cancer [36]. Murine studies about cancer showed that excess of calcitriol favors the stimulation of the metastasis of 4T1 mammary gland carcinoma in BALB/c mice [37]; besides, another study about the effects of calcitriol on immunity in 4T1 tumor-bearing mice showed an enhancement of Th2 response and stimulation of Th17 cell differentiation, with an increased local calcitriol synthesis in M2 tumor-associated macrophages. Thus, in cancer, the calcitriol may intensify the immunosuppression of the tumor niche, contributing to the stimulation of cancer progression [36].

In patients with asthma and chronic obstructive pulmonary disease (COPD), it has been reported that inflammatory processes dysregulate the vitamin D metabolism; these patients before and after a cholecalciferol supplementation showed lower calcidiol serum levels and higher calcitriol/calcidiol ratio; moreover, the patients with asthma presented higher calcitriol levels in comparison to controls. In these diseases, the expression of TNF-*α*, IL-1*β*, and TGF-*β* was increased and these proinflammatory cytokines have been reported to induce expression of the enzymes CYP24A1 and CYP27B1, which are related to calcidiol and calcitriol enzymatic hydroxylation, respectively [38].

Concerning the vitamin D hydroxylation efficiency evaluated in our study by the calcitriol/calcidiol ratio, the SLE patients showed a higher ratio than the HS, representing that SLE patients are converting more calcidiol to calcitriol, like the pattern described in patients with asthma and COPD [38]. In our study, this pattern of high vitamin D hydroxylation efficiency ratio and high calcitriol serum levels was more evidently observed in calcidiol-deficient SLE patients with clinical and renal disease activities. According to our findings, Pasquali et al. reported in subjects with risk to renal disease and calcidiol deficiency a similar pattern of high calcitriol/calcidiol ratio and high calcitriol serum levels; besides conforming that the calcidiol sufficiency was reached, the calcitriol/calcidiol ratio was lower compared to those of calcidiol-deficient subjects [20], highlighting a possible compensatory regulation mechanism that promotes a higher calcitriol synthesis when the calcidiol deficiency is presented.

Regarding this, it has been described that the inflammatory state modulated by TNF-*α* and IFN induces via Toll-like receptors (TLRs) the upregulation of the VDR expression on the macrophage surface, inducing the activity of the CYP27B1 enzyme related to the conversion of calcidiol to calcitriol in situ, which has a costimulatory effect on lymphocyte polarization [5, 24, 39, 40]. This event might support that the immune cells follow a biphasic dose response of several cytokines and nutrients, as suggested for calcitriol concentrations [41]. In a murine model of experimental autoimmune encephalomyelitis (EAE), a moderate diet in vitamin D (1500 IU/kg food) decreased the severity of EAE, while high doses of cholecalciferol supplementation (75000 IU/kg food) enhance activation, proinflammatory differentiation of T cells, and phagocytic activity of peripheral myeloid antigen-presenting cells, characterized by an enhanced surface expression of MHC class II and costimulatory molecules CD40, CD80, and CD86 [42]. Additionally, mice with high vitamin D levels showed in the central nervous system higher IFN-*γ* and IL-17 levels with lower frequency of Treg cells [42, 43]. Hence, the beneficial effect of vitamin D may be achieved at moderate doses.

Some studies have reported that calcitriol administration may prevent strong Th1 responses, whereas its effects on Th2 cells are more complex and not fully elucidated [11, 44, 45]. Also, as previously mentioned, a possible biphasic effect of vitamin D on allergies has been described; vitamin D deficiency may be related to allergic reactions; however, it has been hypothesized that the vitamin D excess leads to an increased allergy risk with a Th2 response predominance and high production of specific IgE against the allergen by the B cells, resulting in an acute inflammatory response [44, 45], maybe modulated by the pattern of high calcitriol when the calcidiol deficiency is presented. Notably, in SLE, a Th2 response and activation of B cells have classically been associated with the pathophysiology of the disease.

The high calcitriol serum levels observed in our study could be promoting an increase in the Th2 cell response in the SLE patients. A subtle balance between Th1 and Th2 cells is provided under normal conditions. Nevertheless, in autoimmunity, a strong Th2 predominance leads to pathologic conditions [45]. Several studies suggest that SLE is a Th2 profile-driven disease [4, 6] characterized by IL-4, IL-5, IL-6, IL-10, and IL-13 cytokines. In murine models, IL-4 promoted the survival of autoreactive B cells and the high expression of IL-5 promotes the proliferation and differential signaling of self-antigen-activated B cell [5]. Regarding IL-6 and IL-10, these cytokines have been associated with high clinical disease activity in SLE, while IL-13 induces the expression of IgE, IL-6, and surface antigen such as CD23, CD71, and MHC II, which together could contribute to the exacerbation of the disease [5].

This pattern of low calcidiol and high calcitriol serum levels observed in our study in SLE patients could be influenced by a high polyclonal B cell activation in active SLE patients. The activation of human B cells induces the CYP27B1 enzyme expression; these cells secrete large amounts of calcitriol, enhancing IL-10 expression from activated B cells more than threefold [11, 46]. According to the biphasic functions of calcitriol, IL-10 is another molecule that has a biphasic function, which could be either protective or pathologic following a concentration gradient; in particular, high IL-10 serum levels in SLE are considered pathogenic. Increased IL-10 production induced by calcitriol may promote the B cell differentiation into plasmablasts, promoting B cells' hyperactivity, autoantibody production, and the antibody class switch to IgA, IgG, and IgM [5, 13, 46, 47].

Notably, in our study, the SLE patients with renal disease activity also showed the same pattern of lower calcidiol serum levels with higher vitamin D hydroxylation ratio and higher calcitriol serum levels in comparison to SLE patients without renal activity. According to this, in SLE patients from Poland, 37% of patients with renal disorder showed lower calcidiol serum levels than patients without renal disorder (*p* = 0.006) [48]. In another study conducted in African-American and Caucasian SLE patients, 18% of patients showed low calcidiol serum levels (<10 ng/mL) and the presence of renal disease was a predictor to lower calcidiol serum levels (OR = 13.3, 95% CI 2.3-76.7, *p* < 0.001) [49], while in pediatric SLE patients from the U.S., the presence of proteinuria (urine protein/creatinine ratio ≥ 0.5) was associated with a decrease of 13 ng/mL of calcidiol and 19.7-fold increased risk of deficient calcidiol serum levels ≤ 10 ng/mL (95% CI 1.8-944.5) [50]. Nevertheless, in these previous studies, calcitriol serum levels were not quantified to be able to contrast the pattern observed in our results.

In our study, high calcitriol/calcidiol ratio scores and high calcitriol serum levels were negatively correlated with the GFR in SLE patients. These observed findings highlight that the kidney is not the only source of calcitriol in patients with renal compromise, is possible that the calcitriol/calcidiol ratio could represent the extrarenal synthesis of calcitriol by immune cells [20]; this event could explain why the SLE patients with renal disease activity had higher calcitriol serum levels in comparison to inactive renal patients.

Regarding this, immune cells such as monocytes, DCs, and B cells can produce calcitriol by the local CYP27B1 expression [45]. Calcitriol enhances the polarization to the Th2 phenotype [11], and the contributions of Th2 cytokines to the renal disease include the production of IL-6 and IL-4 by activated basophils that leads to autoantibody deposition in the kidney via enhanced Th2 response and B cell activation, promoting the release of Th2 cytokines such as IL-10, IL-13, and IL-6; the influx of inflammatory cells; and renal manifestation in active SLE or SLE patients with LN, compared to patients without renal disorders [4, 5].

Therefore, although calcidiol is considered the best indicator of the serum vitamin D status, its deficiency could not reflect calcitriol's serum status [20]. Despite that the half-life of calcitriol in the circulation is very short (12–36 h), its concentration appears to be relatively steady because, after a negative or positive stimulus, several weeks are necessary to return to the prestimulus calcitriol serum range. This indicates that one blood measurement at a specific time point is sufficient to estimate the range in which circulating calcitriol concentrations will be seen over several weeks [44]. Hence, the implementation of the quantification of both vitamin D metabolites, calcidiol and calcitriol, together with the evaluation of the calcitriol/calcidiol ratio, could be important biomarkers in SLE to elucidate in a better way the complex biological regulation of the vitamin D serum status in the clinical disease and renal activities in SLE.

**Figure 6:**
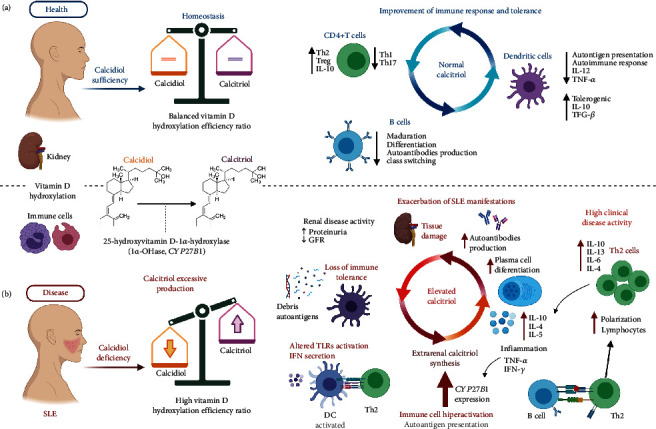
Biphasic effect of calcitriol on health and autoimmunity. (a) In health, the beneficial effect of calcitriol could be achieved at moderate concentrations presented in the calcidiol sufficiency status. Adequate concentrations of calcidiol and calcitriol (vitamin D sufficiency) allow adequate immunomodulation that improves the immune response and tolerance, favoring polarization towards the tolerogenic Th profiles, reducing the maturation of autoreactive B cells and the antigenic presentation of the dendritic cells, with an increase of IL-10 and TGF-*β* cytokines. (b) In SLE, lower calcidiol serum levels (vitamin D deficiency) increase the vitamin D hydroxylation efficiency ratio and the extrarenal calcitriol synthesis; this event may occur due to a compensatory mechanism where the immune cell system in a proinflammatory autoimmune context increases the extrarenal hyperactivity of the CYP27B1 enzyme, contributing to an increase in the calcitriol serum concentrations by calcidiol hydroxylation and promotes the exacerbation of the pathological autoimmune response, characterized by a positive feedback loop of the aberrant self-antigenic presentation, the plasma cell differentiation, autoantibody production, autoreactive Th2 polarization, tolerance loss, and damage to tissues.

Based on our results and previous findings reported about calcitriol, we hypothesized that the pattern of high calcitriol serum levels and increased synthesis of calcitriol in calcidiol-deficient SLE patients could be due to a compensatory mechanism to regulate the low amount of available calcidiol serum, and in an autoimmune context, the increase of calcitriol serum concentration in calcidiol-deficient SLE patients that present clinical and renal activity could contribute to the exacerbation of the autoimmune response. Notably, in physiological conditions, calcitriol's beneficial effects could be achieved at moderate calcitriol concentrations presented in the calcidiol sufficiency status (Figure 6).

According to our results, the strength of the present study was that the sample of SLE patients and HS evaluated was homogeneous in the following characteristics: all participants were female, from the same geographic area (western Mexico), classified as the Mexican-mestizo population with three ancestors in the same geographic region, and of similar age, which reduces the bias regarding environmental and genetic factors of ancestry that could influence the results. Nevertheless, our methodological constraints were that our cross-sectional study design limited us by simply showing an association between high calcitriol serum levels and the calcitriol/calcidiol ratio with SLE and renal disease activities. Therefore, we do not suggest causality because our study only provides information on a specific time point. Moreover, the other limitations of the present study were that we did not quantify the serum levels of proinflammatory cytokine associated with pathogenic Th2 response in SLE and the molecules related to vitamin D metabolism, such as cholecalciferol, PTH, and phosphorus, that could also influence the vitamin D serum levels. Furthermore, to test our hypothesis, we were not able to evaluate the expression at the mRNA, protein level, and enzymatic activity of the vitamin D hydroxylase enzymes involved in the catabolism of calcidiol such as CYP24A1 and the calcitriol renal and extrarenal synthesis such as CYP27B1 in the kidney and leucocytes from SLE patients and HS with vitamin D deficiency.

Therefore, further studies in SLE focused on these points will be necessary to perform, to assess the causality of the relationship of low calcidiol, high-calcidiol-to-calcitriol conversion rate, and high calcitriol serum levels in SLE patients with clinical and renal disease activities described in the present cross-sectional study. These future studies will help to support the clinical and translational interventions with vitamin D supplementation in subsequent studies conducted in patients with autoimmune diseases.

## 5. Conclusions

In conclusion, a pattern of calcidiol deficiency with high calcitriol serum levels and high vitamin D hydroxylation efficiency ratio was associated with disease risk in SLE patients.

## Figures and Tables

**Table 1:** Clinical characteristics by clinical activity in SLE patients.

Variable	Total *n* = 146	SLE patients		*p* value
		Clinical activity (Mex-SLEDAI ≥ 2) *n* = 63	Clinical inactivity (Mex-SLEDAI < 2) *n* = 83	
Age (years)^a^	37 (20–58)	36 (21–56)	37 (20–60)	0.65
*Disease evolution time (years)*				0.44
<5 years^b^	44 (63/143)	48 (30/63)	41 (33/80)	
>5 years^b^	56 (80/143)	52 (33/63)	59 (47/80)	
SLICC ACR-DI^a^	0 (0–4)	1 (0–4)	0 (0–2)	**<0.001**
Damage (SLICC ≥ 1)^b^	42 (55/130)	64 (36/56)	26 (19/74)	**<0.001**
Renal activity ^b^	30 (26/87)	45 (15/33)	20 (11/54)	**0.01**
Renal insufficiency ^b^	14 (12/83)	21 (7/34)	10 (5/49)	0.18
*Serum variables*				
ANAs (+)^b^	81 (100/123)	81 (44/54)	81 (56/69)	0.96
Anti-dsDNA (+)^b^	42 (59/139)	70 (16/23)	53 (20/38)	0.19
C3 (mg/dL)^a^	114 (55.3–150)	101 (1.17–123)	115 (55.3–156)	0.20
C4 (mg/dL)^c^	15.5 ± 8.01	12.7 ± 7.47	17.2 ± 8.09	0.19
Calcidiol (ng/mL)^a^	21.9 (5.1–40.4)	21.2 (3.26–36.6)	22.4 (12.0–40.4)	0.14
*Calcidiol reference values % (n*)				0.34
Sufficiency (≥30 ng/mL)^b^	18 (26/144)	13 (8/62)	22 (18/82)	
Insufficiency (≥20 to <30 ng/mL)^b^	38 (55/144)	39 (24/62)	38 (31/82)	
Deficiency: (<20 ng/mL)^b^	44 (63/144)	48 (30/62)	40 (33/82)	
Calcitriol (pg/mL)^a^	46.9 (22.1–101)	48.5 (21.7–117)	46.2 (22.1–95.2)	0.32
*Calcitriol tertiles % (n*)				0.26
*T*3^rd^ (≥48.7 to 157.3 pg/mL)^b^	43 (61/142)	50 (30/60)	38 (31/82)	
*T*2^nd^ (≥33.6 to <48.7 pg/mL)^b^	37 (53/142)	30 (18/60)	43 (35/82)	
*T*1^st^ (0.33 to <33.6 pg/mL)^b^	20 (28/142)	20 (12/60)	19 (16/82)	
Calcitriol/calcidiol ratio (pg/ng)^a^	2.15 (0.73–9.13)	2.78 (0.84–18)	1.92 (0.71–6.43)	**0.02**
*Calcitriol/calcidiol ratio tertiles % (n*)				0.58
*T*3^rd^ (≥2.23 to 23.6 pg/ng)^b^	47 (67/141)	52 (31/60)	44 (36/81)	
*T*2^nd^ (≥1.36 to ≤2.23 pg/ng)^b^	31 (44/141)	27 (16/60)	35 (28/81)	
*T*1^st^ (0.01 to <1.36 pg/ng)^b^	21 (30/141)	21 (13/60)	21 (17/81)	
Serum calcium (mg/dL)^c^	9.24 ± 0.53	8.94 ± 0.61	9.44 ± 0.35	**0.01**

^a^Data provided in medians (percentile: *p*05^th^–*p*95^th^), Mann–Whitney test. ^b^Data provided in percentages (*n*/total patients), *χ*^2^ test. ^c^Data provided in mean ± SD, Student *t*-test. The bold numbers indicate the variables with significant differences. SLICC: Systemic Lupus International Collaborating Clinics, American College of Rheumatology Damage Index; Mex-SLEDAI: Mexican Systemic Lupus Erythematosus Disease Activity Index; ANAs: antinuclear antibodies; Anti-dsDNA: anti-double-stranded DNA antibodies; C3 and C4: complement.

**Table 2:** Clinical characteristics by renal activity in SLE patients.

Variable	Total *n* = 88	SLE patients		*p* value
		Renal activity *n* = 27	No renal activity *n* = 61	
Age (years)^a^	33 (20–59)	32 (19–58)	33 (20–59)	0.44
*Disease evolution time % (n*)				0.06
*>5 years^b^*	48 (41/85)	33 (9/27)	55 (32/58)	
SLICC ACR-DI^a^	0 (0–04)	1 (0–5)	0 (0–3)	**0.02**
*Damage (SLICC*≥*1)^b^*	39 (29/74)	56 (13/23)	31 (16/51)	**0.04**
Mex-SLEDAI^a^	0 (0–8)	2 (0–8)	0 (0–7)	**<0.01**
Clinical disease activity (≥2)^b^	38 (33/87)	58 (15/26)	30 (18/61)	**0.01**
Renal insufficiency^b^	14 (12/83)	37 (10/27)	4 (2/56)	**<0.001**
*Serum variables*				
ANAs (+)^b^	94 (63/67)	96 (24/25)	93 (39/42)	0.60
Anti-dsDNA (+)^b^	59 (36/61)	82 (18/22)	46 (18/39)	**<0.01**
C3 (mg/dL)^a^	114 (55.3–150)	75 (55.3–120)	115 (1.17–156)	**0.03**
C4 (mg/dL)^c^	15.5 ± 8.01	13.4 ± 9.37	16.3 ± 7.53	0.43
*Calcidiol reference values % (n*)				**0.03**
Sufficiency (≥30 ng/mL)^b^	24 (20/82)	27 (7/26)	21 (13/61)	
Insufficiency (≥20 to <30 ng/mL)^b^	43 (35/82)	23 (6/26)	52 (32/61)	
Deficiency (<20 ng/mL)^b^	33 (27/82)	50 (13/26)	26 (16/61)	
*Calcitriol tertiles % (n*)				**0.03**
*T*3^rd^ (≥48.7 to 157.3 pg/mL)^b^	24 (21/88)	41 (11/27)	16 (10/61)	
*T*2^nd^ (≥33.6 to <48.7 pg/mL)^b^	49 (43/88)	44 (12/27)	51 (31/61)	
*T*1^st^ (0.33 to <33.6 pg/mL)^b^	27 (24/88)	15 (4/27)	33 (20/61)	
*Calcitriol/calcidiol ratio tertiles % (n*)				0.26
*T*3^rd^ (≥2.23 to 23.6 pg/ng)^b^	29 (25/87)	38 (10/26)	25 (15/61)	
*T*2^nd^ (≥1.36 to ≤2.23 pg/ng)^b^	41 (36/87)	42 (11/26)	41 (25/61)	
*T*1^st^ (0.01 to <1.36 pg/ng)^b^	30 (26/87)	19 (5/26)	34 (21/61)	
Serum calcium (mg/dL)^c^	9.25 ± 0.53	9.1 ± 0.90	9.3 ± 0.39	0.43

^a^Data provided in medians (percentile: *p*05^th^–*p*95^th^), Mann–Whitney test. ^b^Data provided in percentages (*n*/total patients), *χ*^2^ test. ^c^Data provided in mean ± SD, Student *t*-test. The bold letters indicate the variables with significant differences. SLICC: Systemic Lupus International Collaborating Clinics, American College of Rheumatology Damage Index; Mex-SLEDAI: Mexican Systemic Lupus Erythematosus Disease Activity Index; ANAs: antinuclear antibodies; Anti-dsDNA: anti-double-stranded DNA antibodies; C3 and C4: complement; GFR: glomerular filtration rate; G1: normal or high; G2: mildly decrease; G3a: mildly to moderately decrease; G3b: moderately to severely decrease; G4: severely decreased; G5: kidney failure.

## Data Availability

The data used to support the findings of this study are available from the corresponding author upon reasonable request.

## Abbreviations

ACR: American College of Rheumatology
GFR: Glomerular filtration rate
HS: Healthy subjects
Mex-SLEDAI: Mexican Systemic Lupus Erythematosus Disease Activity Index
mL: Milliliter
ng: Nanograms
pg: Picograms
SLE: Systemic lupus erythematosus
SLICC-DI: Systemic Lupus International Collaborating Clinics Damage Index
T: Tertile.
